# Supplementary figures and images for: Identification of biomarkers in Alzheimer’s disease and COVID-19 by bioinformatics combining single-cell data analysis and machine learning algorithms
Source: PLoS One. 2025 Feb 18;20(2):e0317915. doi: 10.1371/journal.pone.0317915 (PMC11835241; doi:10.1371/journal.pone.0317915)

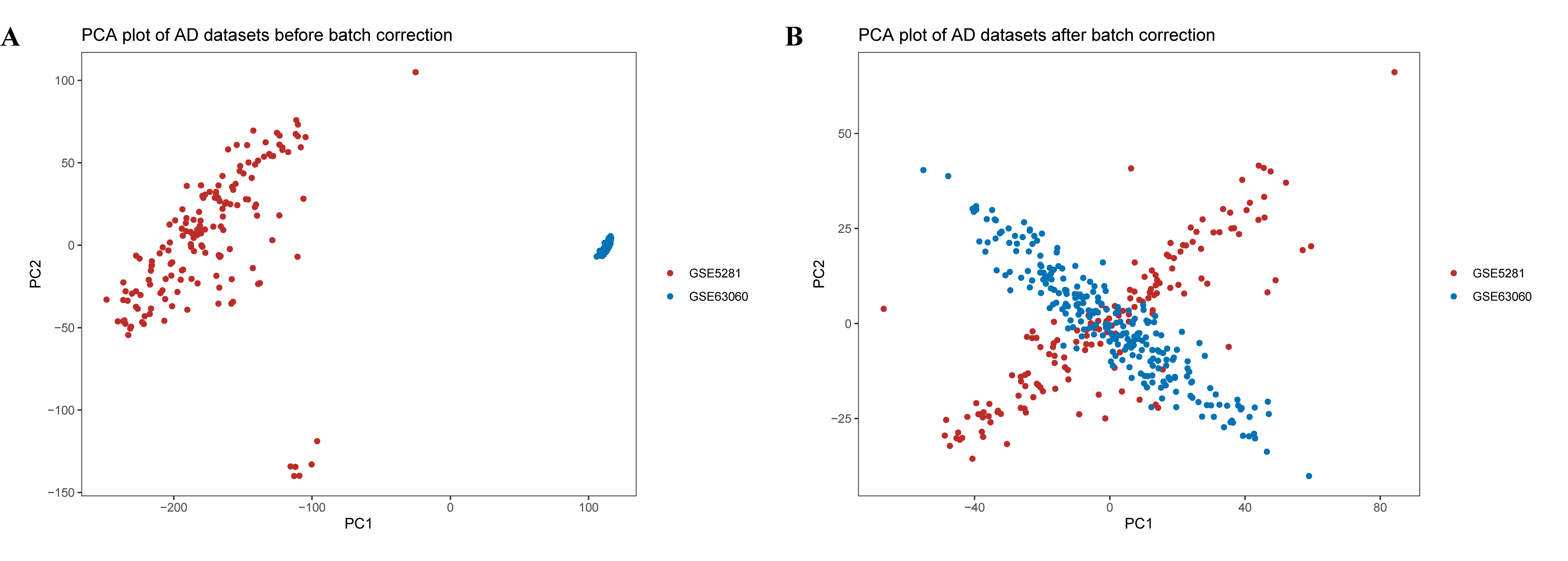

Supplement: S1 Fig — (A) Prior to batch correction, samples from GSE5281 (brain tissue) and GSE63060 (blood) showed clear separation based on tissue origin. (B) After applying ComBat batch correction, there was a significant reduction in strict tissue-based separation, while maintaining biological variability. (TIF) [file pone.0317915.s001.tif]

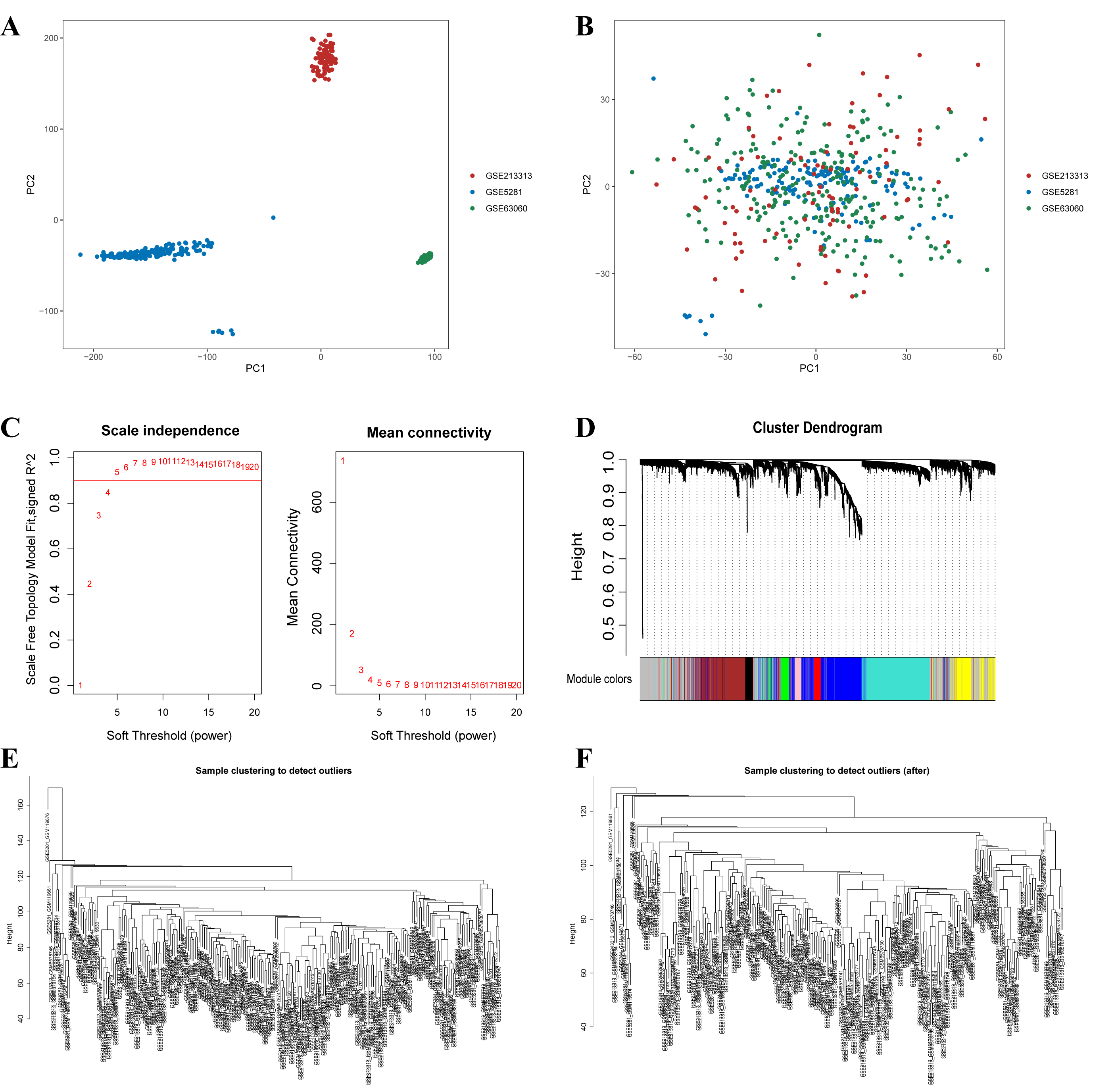

Supplement: S2 Fig — (A) PCA plots of datasets GSE213313, GSE5281 and GSE63060 before de-batching (B) PCA plot of datasets GSE213313, GSE5281 and GSE63060 after de-batching (C) Analysis of the network topology of soft threshold power (D) Cluster dendrogram identifying co-expressed genes (E) Hierarchical clustering before outlier removal (F) Hierarchical clustering after outlier removal (threshold: 140). (TIF) [file pone.0317915.s002.tif]

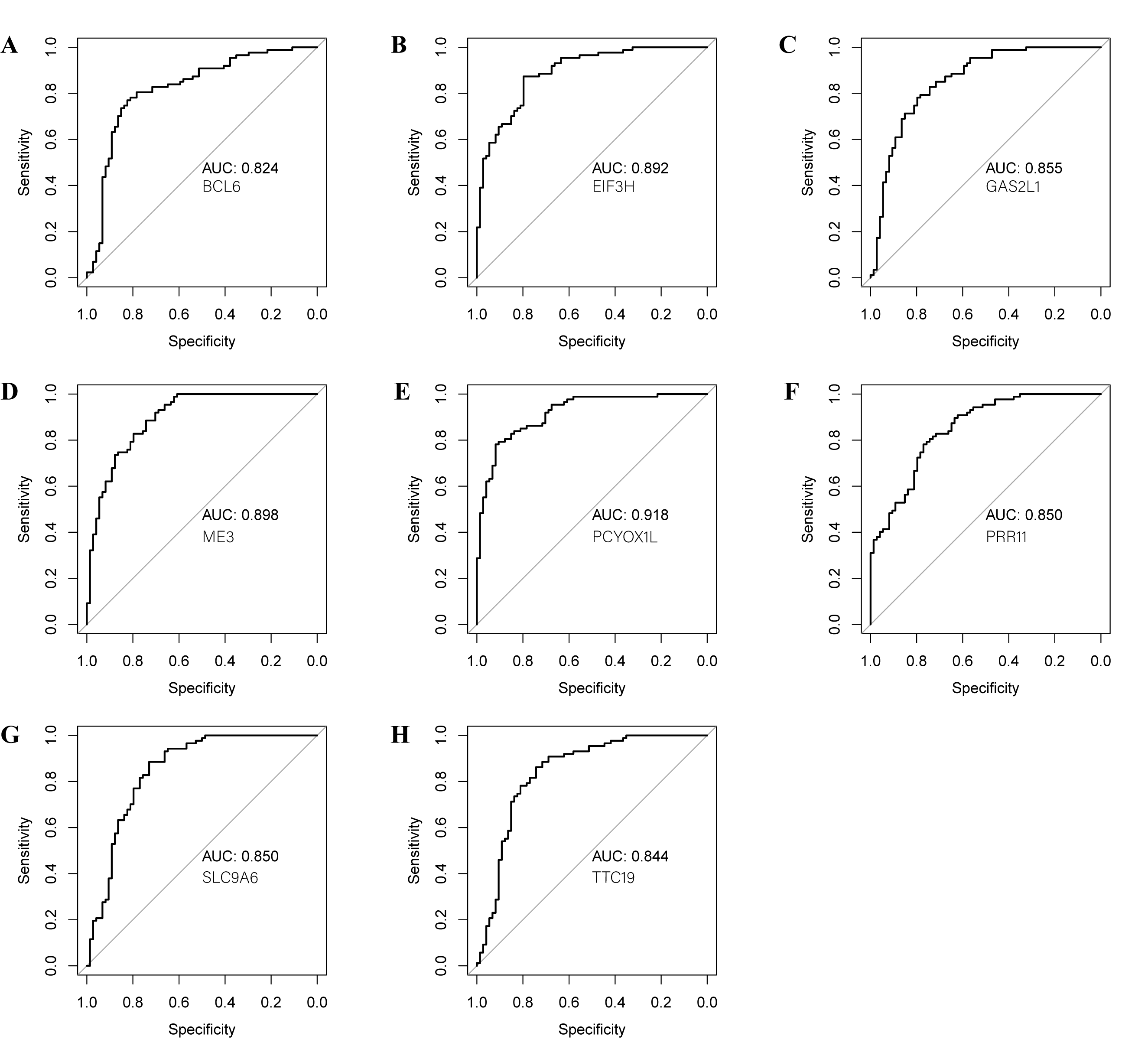

Supplement: S3 Fig — (A) Gene BCL6 (B) Gene EIF3H (C) Gene GAS2L1 (D) Gene ME3 (E) Gene PCYOX1L (F) Gene PRR11 (G) Gene SLC9A6 (H) Gene TTC19. (TIF) [file pone.0317915.s003.tif]
