# Supplementary material for: Identification of biomarkers in Alzheimer’s disease and COVID-19 by bioinformatics combining single-cell data analysis and machine learning algorithms
Source: PLoS One. 2025 Feb 18;20(2):e0317915. doi: 10.1371/journal.pone.0317915 (PMC11835241; doi:10.1371/journal.pone.0317915)
Supplement: Attachment — Submitted filename: Response to Reviewers.docx [file pone.0317915.s004.docx]

Dear Editor,

I would like to express my sincere gratitude for your thoughtful review and invaluable guidance on my manuscript. Your dedication and insights have been instrumental in improving the quality of this work, and I am deeply appreciative of your support. All revisions to the original text, including additions, deletions, and modifications, have been highlighted in red. Firstly, in response to your suggestion “The emerging roles of COVID-19 should be further discussed”, we have explored and discussed in detail the new directions of long-term interaction between COVID-19 and AD, metal ions, mitochondrial dysfunction and oxidative stress in the light of our findings. We have also added the corresponding excellent literature as support.

Additionally, if you request to share the code, our research team is willing to upload and share the entire code involving the study according to the specifications and without reservation.

Below, I provide detailed responses to each of the reviewers' questions.

**For the first reviewer**:

Thank you for your valuable comments and insightful suggestions. We have carefully reviewed each of your points and made the necessary revisions to improve the clarity and quality of our manuscript. Detailed responses to each comment are provided below.

1. **Reviewer’s comment**: The authors should explain all the abbreviations, DEG for exemple…since the first use

**Response**: We are grateful to the reviewer for noting the importance of defining abbreviations upon first use, and we have made the suggested adjustments. We have now ensured that all abbreviations, including “DEG” (differentially expressed genes), are defined upon their first use in the manuscript. This greatly improves the clarity of the text for readers unfamiliar with the relevant terminology.

2. **Reviewer’s comment**: Figure 1, the authors need to precise for panel C &D “common up/down regulated” as maybe common between AD and COVID-19

**Response**: Thank you for pointing out the clarification needed in Figure 1. We have changed the labels in panel C &D to make it clear that “common up/down regulated” refers to genes that are commonly up- or down-regulated between AD and COVID-19. This adjustment will help avoid any potential ambiguity for the reader. This adjustment will help avoid any potential ambiguity for the reader.

3. **Reviewer’s comment**: Figure 1 is written in small letter, as a reader we need to use the zoom in, maybe change the size of the police…

**Response**: Thank you for pointing out that the font is too small. We have modified the relevant font size in the image appropriately, but due to image length issues we are unable to increase it further.

4. **Reviewer’s comment**: Same remark for figure 2, we can not read without using zoom in

**Response**: I would like to express my gratitude once more for your invaluable contribution. As with the preceding comment, the font size of the text in Figure 2 has been adjusted in accordance with the requisite specifications.

5. **Reviewer’s comment**: Figure 2. The authors need to precise which correlation test they used.

**Response**: I am grateful for your recommendations regarding the methodology for conducting correlation tests. In Figure 2, the Pearson correlation test was employed to analyse the correlation between the two diseases. The legend has been modified to explicitly indicate that the Pearson correlation test was used to assess these correlations.

6. **Reviewer’s comment**: The authors need to rephrase “the results revealed that these genes were associated with pathways such as…” it is more likely that the pathways involving cytokines etc…

**Response**: I am grateful for your proposal. In light of the feedback provided, the sentence has been rephrased to clarify that the pathways in question involve biological processes such as cytokines and growth factors, rather than directly associating genes with these molecules. The revised sentence now reads as follows: The results demonstrated that these genes were implicated in pathways associated with cytokines, growth factors, apoptosis, and responses to metal ions and inorganic substances. This amendment enhances the clarity and precision of the biological relationship being delineated. (Lines 146-148)

7. **Reviewer’s comment**: Figure 5 A, the number on the top of the graph should be explained… represented r? p value ? or other?

**Response**: Your comments about providing further explanation of the numbers at the top of the picture are much appreciated. To better explain Figure 5 A, we have added the following note to the results section of the main text: Moreover, there is no significant correlation between both nCount RNA and nFeature RNA and percent.mt (correlation coefficients are -0.05 and -0.04, respectively). And the following description has been added to the figure legends: The values at the top of each panel represent the correlation coefficients between the variables shown on the axes. (Lines 249-251)

8. **Reviewer’s comment**: Figure 6 A and B, the writing on the panels are very small

**Response**: Thank you again for your suggestion regarding the font size in the image. We have adjusted the text size in Figure 6 to enhance readability.

9. **Reviewer’s comment**: The authors need to provide the GEO number of AD and COVID19 data set used, in the first sentence of the Materials and Methods/ Data download, the link refered to the principal webpage…and not in the results part

**Response**: We concur with and are grateful for this recommendation. Consequently, we have amended the wording in the outcome to read: The limma package was utilized to identify differentially expressed genes (DEGs) between the COVID-19 (n = 34) patients and healthy controls (n = 11). And we have incorporated a description of the dataset numbers and URLs in the methods section, as recommended. (Lines 104-105 and 488-493)

10. **Reviewer’s comment**: The authors need to explain the limma package in short sentence…

**Response**: We would like to express our sincerest gratitude for this valuable suggestion. In response, we have provided a comprehensive and informative overview of the “limma” package, emphasising its relevance to our analysis. We have also included references to show why we use this package and where readers can find more information. The objective of these additions is to enhance the clarity and rigour of our methodological approach. (Lines 493-496)

11. **Reviewer’s comment**: The authors should explain their choice for a threshold of 140 to remove the outlier samples.

**Response**: We agree with this comment, which could make the data processing process more understandable. We have therefore added explanations and, in addition, modified some of the descriptions in order to make the textual descriptions more fluent. (Lines 133-135)

12. **Reviewer’s comment**: The authors need to explain why they used beta threshold at 6.

**Response**: We are very grateful for this suggestion. In response, we have provided a more comprehensive explanation of the theoretical basis and rationale behind selecting 6 as the soft threshold power (β). This additional context aims to clarify our choice and enhance the transparency of our methodology. (Lines 511-517)

13. **Reviewer’s comment**: The authors claimed in legend of the figure 7C co-localisation and in the text, association, maybe they need to explain these 2 concepts…

**Response**: Thank you for pointing out the potential confusion between the terms "co-localisation" and "association". Indeed, an association between EIF3H and oxidative phosphorylation was obtained by single-cell localisation of immune cells. However, for the sake of rigour and consistent terminology throughout the paper, we have modified the description here and called it "co-localisation". In the discussion section, we further elaborate on the relationship between co-localisation and association. We initially explored the relationship between EIF3H and oxidative phosphorylation by co-localisation analysis of immune cells. This only reflects the association to a certain extent, which needs to be further explored in related studies, so we have improved the presentation here for rigour. We have also revised the presentation of the methods section to make it more in line with academic norms. Thanks again for this great suggestion, which plays an important role in contributing to ensuring rigour and uniformity in the presentation of our articles. (Lines 277-279 , 416-419 and 550-553)

**For the second reviewer**:

We would like to thank you for your valuable feedback and constructive recommendations. Your comments have been instrumental in improving our work, and we have addressed each point in great detail in the revised manuscript. Please find our detailed responses to your comments below.

1. **Reviewer’s comment**: The work provides a interesting descriptive statistical analysis of comparing genetic regulation in Alzheimer’s disease and covid-19 patients. The presented statistical pipeline leads into a robust model that could be used for exploring relations between inherited and infectious diseases. The work shows a novel approach and robust bioinformatical analysis. In the biological aspect, the work is much more limited. The authors do implore general mechanisms of biology behind covid and Alzheimer’s disease, however they fail to explore the topics in detail and the relation between covid and AD is not fully investigated. The authors should consider discussing possible mid and long-lasting effects of covid-19 on Alzheimer disease patients, including long-covid or PASC symptoms. Authors should also briefly introduce Alzheimer’s disease causes and symptoms in the introduction, as well as explore the genetic causes leading to variable outcomes of severity of both diseases.

**Response**: Thank you very much for your acknowledgement of our analytical methods and model construction work, as well as your valuable comments on the biological mechanisms underlying our extended findings. We strongly agree with these important suggestions you made and have revised the Discussion section at greater length to explore the biological significance of the results of this study in greater depth.

Specifically, we have delved into the biological mechanisms that metal ion homeostasis may play in the pathogenesis and progression of these two diseases. In addition, we have comprehensively elaborated in detail how changes in metal ion levels affect neuroinflammation and oxidative stress, which may play an important role in the interplay between AD and COVID-19, in the context of previously available studies. We have also added consideration of long-term COVID-19 to our discussion of the results, which adds to the length of time uniformity with AD, which is also a chronic course disease, and helps readers to better understand the mechanisms of interaction between these two diseases.

Furthermore, an introduction to the aetiology and symptomatology of Alzheimer's disease has been incorporated into the Introduction section, and an investigation of the genetic factors that contribute to the differing degrees of severity observed in the two diseases has been undertaken. These additions and revisions are intended to enrich the exploration of biological mechanisms and provide a more comprehensive understanding of the potential link between AD and COVID-19.

2. **Reviewer’s comment**: The identification of shared biomarkers with high diagnostic potential could have important implications for the development of co-diagnostic tools and the understanding of the interplay between these two complex diseases. I would welcome a clearer explanation of how this work can prove to be valuable in diagnosis and treatment of diseases, as mentioned on lines 158-159. The work could indeed be very useful in this aspect, and therefore there should be more focus placed on it.

**Response**: We are most grateful for the proposal. The discussion of the actual value of these diagnostic biomarkers is really important. We have further explained and illustrated the practical value of these diagnostic biomarkers in the results section. Corresponding to the description of diagnostic biomarkers in the results section, we have added further discussion of their practical implications, such as therapeutic and drug development target selection and the development of diagnostic tools, based on valuable input. (Lines 195-198 and 371-377)

3. **Reviewer’s comment**: On lines 165-179, The work evaluates data from patients. Is there any information on the disease progression of the patients, perhaps even mortality rates? If there is significant prevalence of covid related complications in one of the groups, probably in the Alzheimer patients, could this effect the outcomes or conclusions of the study? For example, if the AD group exhibited a higher level of mortality, could this be caused by exacerbated immune response that this group is prone to?

**Response**: This insightful comment is much appreciated. Although this data does not provide data on disease progression or mortality in patients, we do not believe that this will have a significant impact on our main results and conclusions. This is because the focus of our analyses was on core genes and immune infiltration and the correlation between them. These analyses were based on gene expression data. The lack of relevant prognostic data may limit our analyses of the association between disease progression and the strength of the immune response, but our analyses were still able to provide valuable insights into immune cell and gene expression in AD and COVID-19. And we have added relevant notes in the Discussion section to explicitly discuss this data limitation and potential implications. (Lines 453-463)

4. **Reviewer’s comment**: Lines 245 to 272 discuss the regulation of interaction of metal ions with genes regulated in covid patients. I find this part slightly lacking credibility. Iron, one of the major metal ions figuring in covid infections is not mentioned at all, furthermore it is well known that metal ion homeostasis is important in other infectious diseases as well. Authors should discuss these two points in more detail. Metal ion homeostasis is common in both AD and covid, however this does not mean that there is a direct link between those occurrences, instead it might be rather a symptom of unrelated origin. Authors should consider whether their results could imply that covid could worsen Alzheimer’s disease progression by altering metal ion homeostasis, which would be a intriguing finding if supported by further studies.

**Response**: We are grateful to the reviewers for their valuable insights into the regulatory processes governing metal ions, particularly iron ions, in the context of COVID-19 and Alzheimer's disease. We acknowledge that the discussion of this section in the previous version of the manuscript was not sufficiently insightful and appreciate the suggestion that we consider the widespread nature of metal ion homeostasis.

In response to the reviewers' comments, the discussion section on the physiological and pathological roles of metal ions has been revised and now includes a discussion of the roles of iron, copper, and zinc ions in relation to immunity and oxidative stress. We elaborated on the important role of metal ions, including iron ions, in COVID-19 and AD.

Furthermore, we have expanded the discussion to emphasise the importance of metal ions, including iron ions, in the context of infectious diseases. We present an insightful discussion of how metal ion dysregulation is a common factor in both diseases and may influence disease course and outcome. Furthermore, we put forth the novel hypothesis that the progression of AD may be influenced by alterations in metal ion homeostasis, which could be a consequence of the effects of SARS-CoV-2 infection. In light of this hypothesis, which offers a novel perspective on the underlying mechanisms, we have revised the manuscript and propose further investigation. It is our contention that this could provide new insights into the pathophysiology of both diseases.

In addition to the comprehensive and systematic elaboration of the role of metal ions in the aetiology of both SARS-CoV-2 infection and AD in the preceding section of the discussion, we further enhance the possible involvement of metal ions with oxidative stress, or the closely related oxidative phosphorylation, in the co-morbid mechanisms of SARS-CoV-2 infection and AD for further elaboration.

We would like to express our gratitude once more to the reviewers for their insightful comments, which have significantly enhanced the quality and credibility of the manuscript and provided valuable guidance for future research directions. (Lines 314-365)

5. **Reviewer’s comment**: Lines 303-304: “after conducting a thorough analysis of immune penetration and metabolic pathways, it was discovered that these diagnostic genes exhibited significant expression levels in various immune cell subtypes and metabolic pathways” is a very strong statement and the analysis and discussion of this topic is only shallow. Therefore, this statement should be altered.

**Response**: I am most grateful for your advice regarding the description of our tone of voice. We concur with your assessment that this is merely an exploratory study and shouldn't use such a heavy tone. Accordingly, the presentation of the section has been revised. We would like to express our gratitude once more for your meticulous examination of our work. (Lines 401-405)

6. **Reviewer’s comment**: Lines 320-321 state that “During our analysis of AD and COVID-19, we identified a clear correlation in the oxidative phosphorylation pathway among certain core genes” This statement is not clearly supported by the data in the work and should be altered to be explorative, not definitive. Clear in vitro or in vivo data would be needed to fully support this statement, which the authors correctly admit throughout the manuscript.

**Response**: We are grateful for your input regarding the expression in question. Indeed, the tone here was not entirely appropriate for this exploratory research, and thus we have modified the expression to be more in line with the nature of our research.

7. **Reviewer’s comment**: The study does not address potential factors, such as age, sex, geographic distribution or severity of diseases of the patients. Can the authors provide some insight into this information?

**Response**: The comments were of great value and we are grateful for them. It is acknowledged that factors such as patients' age, gender, geographic distribution, or disease severity may have an impact on the generalisability of the study results. However, due to data availability and other reasons, these factors were not included in the analysis. We acknowledge the limitations imposed by this objective reason and have provided a detailed account of these limitations in the Discussion section, along with recommendations for future studies. Meanwhile, we will continue to monitor the status of data updates in order to further advance this study. (Lines 465-482)

8. **Reviewer’s comment**: Line 78: the word “infect” should be “infection”?

**Response**: Thank you very much for pointing out the incorrect use of the word “infect” here. You are correct that “infection” should indeed be used here to be more grammatically correct. We have changed this in the manuscript to ensure clarity and accuracy. (Line 77)

9. **Reviewer’s comment**: Line 88-89: The argument that “Mitochondrial dysfunction and oxidative stress contribute to neuronal damage and worsening of Alzheimer's disease” warrants a citation.

**Response**: We greatly appreciate your suggestions for sensible citations to the relevant literature. Indeed citations are needed here for support, so we have inserted appropriate citations (DOI: 10.3969/j.issn.1673-5374.2013.21.009) here to make it more credible. (Lines 88-89)

10. **Reviewer’s comment**: Line 256: In vitro should be in italics

**Response**: Thank you for your suggestion to italicise “In vitro”. In response to the previous suggestion, we have revised this section of the description and the term “In vitro” has been removed. We have checked the full text to ensure that all Latin terminology complies with journal requirements.

11. **Reviewer’s comment**: Figure 1, A and B don’t bear any significant information value. I would suggest removing these parts of figures, since the data is well described on lines 114-117.

**Response**: I am grateful for your valuable input regarding the organisation of Part A and Part B of Figure 1. Our team has considered this suggestion carefully and has reassessed the significance of this section of the figure. It was agreed that the addition of corresponding images, despite being described in detail in the text, would assist readers in comprehending the data processing flow with greater clarity, particularly those who are less familiar with the methods involved. However, should these images be regarded as superfluous, we are prepared to remove this section and modify it as needed to ensure the images remain clear and concise.

**For the third reviewer**:

We would like to express our sincerest gratitude for your meticulous examination of our manuscript and invaluable recommendations. Your constructive suggestions have significantly enhanced the quality of this study and the article itself. We would like to express our sincerest gratitude for your efforts. We have carefully considered each of your suggestions and implemented the requisite changes. Please find below a comprehensive response to each of your suggestions.

1. **Reviewer’s comment**: The question addressed in this manuscript is important in the field of Alzheimer’s disease research since inflammatory insults like COVID-19 infection likely impact innate immunity which appears to become dysregulated in Alzheimer’s disease. The authors try to pin-point what molecular, metabolic or immune biomarkers are shared between COVID-19 infection and Alzheimer’s disease which would further support a possible link between the two conditions. However, they attempt to do this by making an incompatible comparison between microarray RNA sequencing from blood samples of COVID-19 patients with brain samples from Alzheimer’s patients in search for common biomarkers. Their analyses are impressive and could prove to be informative, but the implications of their findings are diminished by the incompatible comparison (and integration of datasets) without any proof that the datasets are indeed comparable (i.e., with a PCA plot to show similarity among samples from different types of biological samples and datasets).

**Response**: I am most grateful to you for providing these comments. We would like to express our gratitude for your acknowledgement of the significance of the influence of inflammatory insults, such as that caused by the SARS-CoV-2 virus, on innate immunity, particularly within the context of Alzheimer's disease research. It is acknowledged that there is a discrepancy in the tissue sources of the study samples used for the two diseases, which may give rise to concerns. Nevertheless, following a comprehensive review, we maintain that the findings of our study warrant consideration. Furthermore, the manuscript has been revised to provide a more comprehensive analysis of the limitations and potential implications of the data employed, with this information now included in the discussion section. And we propose a cautious approach to the interpretation of the results and suggest potential avenues for future research. The following section presents a comprehensive response to each of the recommendations.

2. **Reviewer’s comment**: The integration of the Alzheimer disease datasets is inappropriate since they were sequenced using different microarray platforms that have been reported to be non-comparable (see https://link.springer.com/article/10.1186/1745-6150-3-23). To find DEGs that are differentially regulated in AD using both datasets, it is recommended that a differential gene expression analysis is completed independently for each dataset, then the common down/upregulated genes can be compared to the COVID dataset.

**Response**: We very much understand the reviewers' concerns about the differences in the microarray platforms we used. We have carefully read this study you refer to. But since the publication of this study, which dates back to 2008, cross-platform data analysis methods have evolved significantly. We use ComBat's approach in our processing of cross-platform data^[1]^. The original article on this method has been cited 6,600 times, which speaks volumes about the reliability and practicability of the method. This method is based on empirical Bayesian statistics, which can well integrate data including, but not limited to, gene chips and other data from different platforms, and is able to deal with the variation of data from different platforms well^[2]^.

In addition, we use the integration results we obtained to do further analyses and find that the results are relatively reliable. Specifically, we obtained some core genes that have been found to play a role in AD and COVID-19 in previous studies, as well as ‘cellular response to metal ions’ and ‘oxidative stress’ pathways, which were confirmed to be closely related to these two diseases. These results also indirectly justify the data processing methods we used. Thus, our method of reliable data integration of cross-platform data is reasonable and rigorous. I would like to express my gratitude once more for your insightful remarks.

3. **Reviewer’s comment**: Based on the heatmap resulting from their WGCNA analysis in Figure 2C, it appears as though the COVID dataset was combined with the AD datasets for the WGCNA analysis. This is also inappropriate unless the datasets were pre-processed (i.e., individual normalization to sample size factor within each dataset) and including a batch-correction. Since they did not clearly mention of how datasets were normalized or genes were filtered, I assume they combined raw sequence counts for all three datasets for their WGCNA analysis. Furthermore, combining the COVID RNAseq from whole blood with AD RNAseq from various brain regions in this WGCNA analysis is less informative since the gene modules likely reflect tissue-specific expression patterns, not disease-related gene signatures.

**Response**: I am most grateful for your valuable suggestions. We are grateful for your attention to the handling of data during the WGCNA process. Furthermore, it is acknowledged that the aforementioned processing was not sufficiently detailed in the previous version of the manuscript. Indeed, each dataset was normalised using the normalizeBetweenArrays function in the limma package, and batch effects were corrected using the ComBat method. As demonstrated by principal component analysis (PCA), the corrected data set exhibits no discernible batch effect. The aforementioned process is illustrated in Fig.S1 and a detailed description is provided in the Methods section.

In regard to the matter of cross-tissue analysis of blood and brain tissue samples, we acknowledge the significance of this issue and offer a plausible explanation as follows:

1) Biological principles:
a) It has been shown that histopathological changes in the brain in AD can be reflected by biomarkers in the blood^[3]^.
b) Shared inflammatory markers exist in the CNS and blood and have been confirmed by studies in AD and COVID-19^[4]^.
c) Both COVID-19 and AD may increase blood-brain barrier permeability, suggesting that there may be a molecular exchange between brain tissue and blood in the context of both diseases^[5, 6]^.
2) Data analysis strategy:
a) Three high volume datasets obtained from Gene Expression Omnibus (GEO) database were used in this study. The datasets used in this study were GSE213313, GSE5281 and GSE63060. the first of these datasets included whole blood transcriptome data from patients diagnosed with COVID-19 and healthy controls. The last two datasets are from AD patients and healthy controls with specific tissue sources of brain and blood, respectively. And through the ComBat method, every effort is made to remove organisational batch differences. The selected samples were all subjected to gene chip technology, which ensured consistency in the level and depth of sequencing, thus guaranteeing comparability between samples.
b) Much to our surprise, the black module was significantly associated with both diseases, despite the fact that the tissue sources of the two diseases were not identical. This association, suggests that the biological relevance has far outweighed the tissue-specific effects.
3) Supporting evidence in the results:
a) We found significant overlap of core biomarkers with previous studies.
b) And further studies have shown that metal ions, oxidative stress, etc. play an important role in both diseases, which has been confirmed by a considerable number of studies. We have elaborated on the above in the Discussion section of the revised manuscript.
4) Clinical Implications:
a) Provides direction for the development of diagnostic markers and treatments common to both diseases.

In summary, we believe that although some tissue variability exists, our rigorous analytical approach enables us to identify associations that go beyond tissue specificity. And there exists a strong explanation based on biological principles and strong corroboration of the results. Therefore, we believe that this WGCNA analysis is still of more significance.

4. **Reviewer’s comment**: There is no discussion about how comparing blood versus brain samples could lead to gene overlap (or exclusions) that are not relevant to the diseases they investigate.

**Response**: We acknowledge and appreciate the reviewer's inquiry regarding the tissue-specific differences between blood and brain samples. A corresponding section has been added to the Discussion section to clarify the cross-tissue analysis of the study and to indicate potential issues with it. As previously stated in response to the last suggestion, we have employed data processing techniques to eliminate tissue-specific effects and have provided authoritative literature to illustrate the close association between the two tissues in both diseases. Furthermore, the biological significance of the results serves to reinforce the soundness of our data processing. In conclusion, we would like to thank the reviewers for their insightful comment. We have compelling reasons to believe in the soundness and scientific validity of the data processing methods employed and have incorporated a discussion of the limitations of the study, as suggested. (Lines 452-481)

5. **Reviewer’s comment**: The authors should have shown if control samples from the different datasets were indeed comparable (i.e., with a PCA plot to show similarity among samples from the three datasets). If the control samples were dissimilar, then one cannot compare/overlap differentially expressed genes between datasets.

**Response**: We would like to express our sincerest gratitude for your invaluable insights pertaining to the comparability of samples. Following a period of reflection and analysis, the research team will now proceed to present a comprehensive account of the data analysis strategy.

Due to the technical differences inherent to cross-platform analyses during data integration, a number of data processing methods were employed to normalise the data in each dataset independently. Subsequently, we concentrated on the common differential genes across the datasets, thereby ensuring more reliable results.

The data analysis strategy enabled the identification of an important metabolic pathway, 'oxidative phosphorylation', and multiple enrichment pathways associated with metal ions. These findings align with those of previous studies, which have demonstrated the crucial roles played by metal ions in the diseases under investigation. The details of these findings are discussed in the Discussion section of the article. Our approach has revealed important biological associations.

In conclusion, we believe that this analysis continues to be of great value and significance. Thanks again for your careful review.

6. **Reviewer’s comment**: More information about gene ontology analysis is required since various tools and pipelines have different algorithms for determining enrichment of specific domains.

**Response**: Thank you very much for your interest regarding the analysis tools and annotation databases used in our gene ontology. This is indeed an important issue that needs to be addressed in the methods section of the article. As described in our new revision in the main text, we used the R package clusterProfiler and the org.Hs.eg.db annotation database in this process. We are grateful for your insights and the description you provided, which we have incorporated into the article in response to your suggestion. The methodological description is now more transparent, which helps readers to understand the tools used and the analysis process. (Lines 501-504)

7. **Reviewer’s comment**: Parameters of the WGCNA pipeline drastically impact the modules so it is important to include exactly what the parameters were (instead of just stating the “optimal” parameter setting was used).

**Response**: We are most grateful to you for this valuable suggestion. In accordance with the aforementioned suggestion, we have provided a concise rationale for the soft threshold (β) selection in the article's methods section and have incorporated pertinent methodological literature. This serves to further substantiate the scientific validity and rigour of this study. (Lines 507-520)

8. **Reviewer’s comment**: Showing the graphical output of intermediate WGCNA steps is unnecessary (and uncommon) but the information from this output (like the optimal soft threshold power that is indicated by the horizontal red line in Figure 2A) should be included in the Methods section.

**Response**: We appreciate your suggestions regarding the WGCNA intermediate steps, which were very professional. We have adjusted the graphics of some of the intermediate steps to the Supplementary Material section based on your suggestions. And the methods section of the WGCNA has been modified to optimise the methodological description of this section and to describe the output information and some important parameters in detail. This has allowed the methods used in this study to be presented in more detail in the article, and has simplified the content of the results images so that important results content is more prominently displayed. (Lines 507-520)

**References**

[1]	Johnson WE, Li C, Rabinovic A. Adjusting batch effects in microarray expression data using empirical Bayes methods. Biostatistics. 2007. 8(1): 118-27.

[2]	Luo J, Schumacher M, Scherer A, et al. A comparison of batch effect removal methods for enhancement of prediction performance using MAQC-II microarray gene expression data. Pharmacogenomics J. 2010. 10(4): 278-91.

[3]	Zetterberg H, Blennow K. Moving fluid biomarkers for Alzheimer's disease from research tools to routine clinical diagnostics. Mol Neurodegener. 2021. 16(1): 10.

[4]	Pomilio AB, Vitale AA, Lazarowski AJ. COVID-19 and Alzheimer's Disease: Neuroinflammation, Oxidative Stress, Ferroptosis, and Mechanisms Involved. Curr Med Chem. 2023. 30(35): 3993-4031.

[5]	Storck SE, Hartz A, Pietrzik CU. The Blood-Brain Barrier in Alzheimer's Disease. Handb Exp Pharmacol. 2022. 273: 247-266.

[6]	Kempuraj D, Aenlle KK, Cohen J, et al. COVID-19 and Long COVID: Disruption of the Neurovascular Unit, Blood-Brain Barrier, and Tight Junctions. Neuroscientist. 2024. 30(4): 421-439.
