# Supplementary material for: Identification of biomarkers in Alzheimer’s disease and COVID-19 by bioinformatics combining single-cell data analysis and machine learning algorithms
Source: PLoS One. 2025 Feb 18;20(2):e0317915. doi: 10.1371/journal.pone.0317915 (PMC11835241; doi:10.1371/journal.pone.0317915)
Supplement: Attachment — Submitted filename: Response to Reviewers.docx [file pone.0317915.s005.docx]

Dear Editor,

I am grateful for your meticulous examination and invaluable counsel regarding our latest revised manuscript. Your expert feedback has been instrumental in further enhancing the manuscript. On behalf of our research team, I would like to express our sincerest gratitude to you. All changes to the previous version of the manuscript, including additions, deletions and modifications, are indicated by red text. In this response, the number of lines is indicated according to the document ‘Revised Manuscript with Track Changes’.

We have firstly revised “the emerging reports about COVID-19” based on your valuable comments. The discussion and citation of the emerging reports about COVID-19 have been incorporated into the preface and discussion sections.

Furthermore, the following three papers, which we consider to be of particular note, have been referenced in lines 61, 465, and 106.

DOI: 10.2174/1573412918666220509032754

DOI: 10.14218/JCTH.2022.00339

DOI: 10.14218/JCTH.2022.00038

Thank you for your consideration.

With kind regards,

Yours sincerely,

Juntu Li

Jun Liu

Department of Critical Care Medicine and Emergency, The Affiliated Suzhou Hospital of Nanjing Medical University (Suzhou Municipal Hospital), Gusu School, Nanjing Medical University, Suzhou Clinical Medical Center of Critical Care Medicine,

No. 16, West Baita Road, Suzhou, Jiangsu Province, China 215001

E-mail: liujunphd@sina.cn

The following section presents a comprehensive response to each of the reviewers' queries.

**For the second reviewer**:

We sincerely appreciate your valuable feedback. We have carefully addressed all your comments and revised our manuscript accordingly. These changes have substantially improved the quality of our work. Below, we provide point-by-point responses to your comments.

1. **Reviewer’s comment**: Minor correction: In several places of the revised manuscript, there is a repeated error in the word "COIVD". Should Be COVID. Lines 196, 458, 460, 474.

The authors addressed my comments and modified the manuscript accordingly. Although I still find some of the discussion regarding biological mechanisms limited, as the authors correctly state, that is not the main focus of the presented work.

In general I find the work robust, exploring a novel and interesting area with a positive impact to the scientific community.

After correcting grammatical errors, I recommend the work for publication.

**Response**: We would like to express our gratitude for your attention to detail in correcting the spelling of certain words in our previous version of the manuscript. The requisite corrections have been made (lines 207, 505, 508, and 521), and the vocabulary has been meticulously examined in order to guarantee the correct spelling.

We appreciate your continued support in improving this manuscript.

**For the third reviewer**:

We greatly appreciate your thorough review and constructive feedback regarding our methodology. In response, we have substantially expanded the Methods section to provide comprehensive details about our analytical pipeline. Please find below a comprehensive response to each of your suggestions.

1. **Reviewer’s comment**: It is unclear what samples from each dataset were used. The AD datasets were combined to result in 232 Alzheimer's patients and 178 healthy controls, but there are more than 410 cumulative samples in those datasets so a subset of samples must have been selected.

**Response**: I am grateful for your interest in the topic of sample size following the merging of our datasets. The AD patient group from dataset GSE5281 comprises 87 samples, while the healthy control group comprises 74 samples. Dataset GSE63060 includes 145 samples from the AD patient group, 104 samples from the healthy control group, and 80 samples from the Mild Cognitive Impairment (MCI) patient group. The complete AD patient and healthy control groups from the aforementioned datasets were included in the analysis. As MCI is not a component of AD, it was not included in our study. Furthermore, a comprehensive account of the methodology employed is provided in the Methods section of the article (lines 560-562), with the aim of offering the reader a more nuanced comprehension of the research data utilised.

2. **Reviewer’s comment**: Typos remain throughout the manuscript.

**Response**: I would like to express my gratitude for your attention to the spelling issue in our manuscript. The manuscript has been subjected to a rigorous spell-checking process, resulting in the correction of some errors, including the replacement of the erroneous “COIVD-19” with the correct “COVID-19” (lines 207, 505, 508, and 521). This makes the article more rigorous and brings it in line with the standard requirements for publication in this journal.

3. **Reviewer’s comment**: Although the authors mention that the limma package “…has powerful read, normalization, and data exploration capabilities,” they should include more precise methodology regarding the normalization of and batch-correction between the datasets. I suggest they add in the details they outline in their response to Reviewer #3’s third and fifth comment into their methods.

**Response**: Thank you for this valuable suggestion to include a more accurate method for normalising and batch correcting datasets. We have already specified this in the previous version of the response letter. We very much agree to include a detailed description of this in the methodology section of the manuscript. We have condensed and distilled it and included it in the main text in a form more suitable for description in the text (lines 577-584).

4. **Reviewer’s comment**: They should include discussion of how the biomarkers they identify overlap/compare with previous studies (and cite those studies) as they suggest they do in the response (3A) to reviewer #3’s third comment.

**Response**: Thank you for your valuable advice. We have partially discussed the overlap of these biomarkers with previous studies in the discussion section of the previous manuscript. Based on your constructive comments, we have further explored in depth the overlap of certain biomarkers with previous relevant studies. In particular, we are notable for BCL6, a gene involved in the pathogenesis of both diseases we have studied (lines 401-415). And appropriate citations have been added. This contributes significantly to the richness of our discussion section.

5. **Reviewer’s comment:** The methods and datasets used to calculate the positive predictive value of these eight identified genes are missing completely. AUC is undefined. Were the ROC curves based on blood samples only (these details need to be included in the methods)? If not, then I suggest re-running these analyses on blood samples only to claim that these genes are potential diagnostic markers. Also, it appears as though the ROC curves are based on presence/absence of COVID and AD (is that correct?). The authors should show the predictability of the eight genes on each disease alone since it would provide insight into whether some genes are more predictive of AD or COVID diagnosis, or if they are specific to the co-diagnosis.

**Response**: Thank you for your suggestions regarding our ROC analysis. We appreciate your careful review and would like to clarify several points:

First, we confirm that our ROC curves were indeed based on blood samples only, considering the technical variations and tissue heterogeneity. We have now revised the methods section to provide more detailed description of our analysis approach (lines 615-618).

Regarding your suggestion about analyzing the predictive power of these genes for each disease separately, we appreciate this perspective. However, our study specifically aimed to investigate the shared molecular signatures between AD and COVID-19, for several key reasons:

1) These eight genes were identified through an integrated analysis pipeline (combining differential expression analysis, WGCNA, and machine learning methods) specifically designed to detect shared molecular features and common pathways between the two diseases. Therefore, our ROC analysis was intentionally focused on validating their collective predictive power for these shared pathological processes.
2) Growing evidence suggests potential molecular links between COVID-19 and AD. Our study aimed to investigate these specific connections, using ROC analysis to validate these shared molecular signatures rather than identifying disease-specific diagnostic markers. This approach is consistent with the findings of recent studies that suggest a potential influence of COVID-19 on the molecular pathology of AD^[1]^.
3) The high AUC values we obtained demonstrate the robustness of these genes as indicators of shared pathological processes between these conditions. This finding supports our research objective of understanding the molecular intersection between AD and COVID-19, which could be particularly valuable for understanding the neurological implications of COVID-19 in the context of AD.
6. **Reviewer’s comment**: Was the AD immune infiltration analysis conducted on composite (blood+brain) dataset? If so, was the analysis on done on the ComBat normalized composite dataset? I ask because I imagine cross-tissue normalization could impact the ability/accuracy of a pipeline like CIBERSORT to determine cell-types within those normalized datasets. In the methods, the authors should add in what datasets were used and how data was handled prior to the CIBERSORT analysis.

**Response**: Expressions of gratitude are extended for the valuable suggestions provided. The CIBERSORT analysis is grounded in a standardised dataset, a practice that ensures the comparability of samples and maintains their biological relevance. In light of the suggestions, deliberations have been held regarding the potential ramifications of data standardisation on ensuing analyses.

Firstly, the CIBERSORT algorithm employs the LM22 feature matrix, which is utilised for immune cell deconvolution. The LM22 contains expression features for 22 immune cell types, including T-cells, B-cells, NK-cells, and so forth, and is a well-proven reference^[2]^. Irrespective of the data preprocessing method employed, this standardised reference matrix enables accurate immune cell proportion estimation^[3]^. The rationale behind this is twofold. Firstly, the algorithm compares relative gene expression values between the sample and the reference matrix, rather than absolute values. Secondly, the algorithm is implemented in a way that focuses on the expression characteristics of the immune cell types that are present in our standardised processed data. This is advantageous because the data we used has a clear distinction between disease and control groups.

While acknowledging the value of tissue-specific analyses as a analytical strategy, our integrative approach offers enhanced access to the comprehensive array of immune pattern alterations in cases of COVID-19 compared to AD. This is a crucial aspect for initial exploration of the link between the two in terms of pathological mechanisms. In response to the recommendations, we have expanded the Methods section of the manuscript to include a detailed description of the utilized datasets and the employed data preprocessing methods (lines 628-632). This has significantly enhanced the clarity of the methods section of the article.

7. **Reviewer’s comment**: Although the authors have revised the discussion to explicitly state that there are limitations due to cross-tissue analyses, I do not agree with the authors in that they have provided “a more comprehensive analysis of the limitations and potential implications of the data…” since they only mention:

521-525: “Furthermore, the cross-tissue nature of the analysis of AD derived from brain tissue versus that of the analysis of COIVD-19 [**typo error] derived from blood may potentially impact the accuracy of the results due to tissue-specific factors. However, we endeavoured to minimise the influence of tissue specificity through the utilisation of the aforementioned methods.”

Only stating that they “endeavoured to minimise the influence of tissue specificity…” is insufficient in The limitations should be discussed in more detail to include discussion of potential non-disease-specific influences (i.e., due to their cross-tissue integration) and why their main findings are likely disease- and not tissue-specific. Additionally, the implications of their findings would be bolstered by discussion of whether (or more so why) they think altered expression of the eight identified genes reflect brain-specific, blood cell-specific or shared mechanisms between tissues (based on what is known tissue-specific gene expression and cell-type-specific functions of their eight identified genes; some of their ideas are included in the response to comment #3). Lastly, the methodology used to minimize tissue-specific influences is not detailed (what are the “aforementioned methods” [is it ComBat?] and is there prior proof that they minimize tissue-specific effects thus allowing for identification of trait-relevant mechanisms?).

**Response**: We would like to express our sincere gratitude for the insightful suggestions you have provided with regard to the tissue specificity and function of the genes outlined in our manuscript. It is acknowledged that the preceding version of the manuscript lacked the requisite level of detail, particularly in addressing the cross-tissue analysis limitations and the potential implications of tissue-specific factors. In light of the constructive feedback provided, substantial revisions have been made to comprehensively address the concerns raised. Specifically, the following changes have been made:

1) Enhanced the discussion of cross-tissue analysis validity from biological perspectives, including evidence of blood-brain barrier permeability and shared inflammatory markers between CNS and blood in both diseases (lines 524-531).
2) Clarified our methodological approach, particularly the use of ComBat method to eliminate tissue batch effects, and explained how gene chip technology ensures sample comparability (lines 531-539).
3) Provided an in-depth analysis of BCL6 as a key example, demonstrating how its involvement in both diseases through conserved molecular pathways represents true disease mechanisms rather than tissue-specific effects (lines 401-415).

In this revision, a detailed discussion on the sharing of genes between different tissues has also been provided. This discussion is supported by relevant literature which demonstrates the biological basis for cross-tissue biomarker studies. The content has undergone a thorough revision process that involved the integration of select components from the prior response to the reviewers. In addition, the description has been revised in the light of the update and the "method" mentioned in the previous manuscript has been clarified in the description.

The incorporation of constructive feedback, our deliberations, and the revision of this section of the content have culminated in a more lucid description of the manuscript, a more cogent discussion, and an enhanced credibility of the results. It is our conviction that these revisions have considerably strengthened the scientific rigor of our cross-tissue analysis.

8. **Reviewer’s comment**: At line 120, the authors have removed details about the bulk RNA sequencing data that was analyzed (removed: “…we acquired and integrated the Alzheimer's disease datasets GSE5281 and GSE63060 (based on platforms GPL570 and GPL21185, respectively)” and replaced it with “…we acquired and integrated two AD datasets”) which decreases the detail necessary to understand that two RNA-seq datasets from two different tissues (brain and blood) that were acquired using different platforms were integrated then analyzed as a single dataset. After further review of current methods for this type of integration, I agree with the authors in that this type of integration can be done and prove to be informative. However, the integration of datasets is complex and non-trivial, so the authors need to detail their integration method which remains missing in the Methods section (i.e., no mention of the ComBat approach). Furthermore, the authors do not perform brain-specific analyses, so it is not apparent why they needed to combine a brain and blood AD dataset to begin with.

**Response**: We really appreciate your helpful suggestions about our dataset description and preprocessing methods. We have addressed each of these suggestions individually:

1) It has been noted that the alterations made to the Methods section have resulted in a reduction of the description of the organisational origin of the dataset, thereby creating a barrier to the comprehension of this study. To address this concern, we have augmented the section pertaining to the organisational sources (lines 555-562).
2) The integration of data is indeed a complex procedure, and we therefore provide a detailed description of this preprocessing procedure, with particular reference to the application of the ComBat methodology used and its role in the elimination of batch effects and tissue-specific variability (lines 577-584).
3) With regard to the integration of brain and blood data for the AD dataset, we have specifically analysed the disease in terms of the primary site of disease, systematic variations, and the possibility that combining the two tissues can provide a more comprehensive understanding of the disease. This is systematically described in detail in the Methods section of the manuscript. This section of the manuscript meticulously delineates the strategic considerations that underpinned our decision to integrate brain tissue and blood datasets for the present study (lines 569-576).

9. **Reviewer’s comment**: The authors do however did satisfy the suggestion to show that the datasets can be well-integrated for the WGCNA (with the PCA plots in Supp Fig 1), but they did not provide proof of the equivalent analysis for the integration of the two AD datasets alone (which was done for the DEG analysis).

**Response**: We are grateful for your constructive proposal regarding the necessity of validating data integration processes. PCA analyses were performed on two AD datasets, derived from two distinct tissue sources, prior to and following correction. The findings have been appended to the appendix images (S1 Fig.). And the legend has been revised (lines 665-670). These results demonstrate that: Prior to correction, the samples exhibited clear clustering according to tissue of origin, signifying a discernible batch effect and tissue specificity. And a corresponding description has been added to the Methods and the Results section of the manuscript (lines 577-584 and 116-121).

Following the correction of these batch effects using the ComBat method, while some biological variation between samples remained, the strict separation between tissues was significantly reduced, suggesting that the batch effects and tissue specificity had been successfully removed, while also ensuring the retention of meaningful biological signals. This additional result provides a justification for the data preprocessing for DEG analysis, allowing for more rigorous data analysis.

10. **Reviewer’s comment**: ‘ssGSEA’ is not defined and the methods they used for determining enrichment via ssGSEA are missing. Are the scale bars/heatmap colors for Fig 4E & 4F representing the correlation coefficient? The authors should explicitly state this in the figure legend. Also, in Figure 4’s legend, it says “Correlation between the expression levels of seven [**Typo? Should be eight?**] hub genes and the ssGSEA enrichment scores…”.

**Response**: We are immensely grateful for these invaluable comments. The following corrections have been made:

1) A detailed description of the ssGSEA method has been added to the Methods section of the manuscript, elucidating the calculation of enrichment scores for each sample (lines 634-638).
2) In the figure legend, it was clarified that the heatmap colours represent the correlation coefficients between the central gene and the ssGSEA enrichment score (lines 241-245).
3) An error in the legend of Figure 4 has been rectified by replacing '7' genes with '8' genes, and the format has been changed to Arabic numerals for consistency (lines 237-239).

These corrections have significantly enhanced the accuracy and clarity of the manuscript.

11. **Reviewer’s comment**: Since the authors are arguing that the differential expression of the eight identified genes are shared between COVID and AD, the authors should reorganize the Figure 4B, 4D, 4E and 4F so that the genes and pathways are in the same order in each figure panel to allow readers to more readily evaluate the similarity of gene and immune cell abundance or pathway correlations in AD and COVID.

**Response**: We are indebted to you for your suggestion to reorganise the order of genes and pathways in Figures 4B, 4D, 4E and 4F. We have reorganised the order of genes, pathways and immune cell types in these panels for the sake of consistency. This arrangement enables the reader to more easily assess and compare the correlation of gene expression patterns with immune cell abundance and pathways between AD andCOVID-19. This modification has resulted in a significant enhancement in the clarity of the shared molecular and immune mechanisms between the two diseases.

**References**

[1]	Jiang Q, Li G, Wang H, et al. SARS-CoV-2 spike S1 protein induces microglial NLRP3-dependent neuroinflammation and cognitive impairment in mice. Exp Neurol. 2025. 383: 115020.

[2]	Gentles AJ, Newman AM, Liu CL, et al. The prognostic landscape of genes and infiltrating immune cells across human cancers. Nat Med. 2015. 21(8): 938-945.

[3]	Newman AM, Liu CL, Green MR, et al. Robust enumeration of cell subsets from tissue expression profiles. Nat Methods. 2015. 12(5): 453-7.
